# Abnormal Osteoblastic Response to Leptin in Patients with Adolescent Idiopathic Scoliosis

**DOI:** 10.1038/s41598-019-53757-3

**Published:** 2019-11-20

**Authors:** Gene Chi-Wai Man, Elisa Man-Shan Tam, Yi Shun Wong, Vivian Wing-Ying Hung, Zongshan Hu, Tsz Ping Lam, Zhen Liu, Wing Hoi Cheung, Tzi Bun Ng, Zezhang Zhu, Yong Qiu, Jack Chun-Yiu Cheng

**Affiliations:** 1 SH Ho Scoliosis Research Laboratory, Department of Orthopaedics and Traumatology, Faculty of Medicine, The Chinese University of Hong Kong, The Prince of Wales Hospital, Hong Kong, SAR China; 2 School of Biomedical Sciences, Faculty of Medicine, The Chinese University of Hong Kong, Hong Kong, SAR China; 3 Department of Spine Surgery, The Affiliated Drum Tower Hospital of Nanjing University Medical School, Nanjing, 210008 China; 4 Bone Quality and Health Centre, Department of Orthopaedics and Traumatology, The Chinese University of Hong Kong, Hong Kong, SAR China; 5 The Joint Scoliosis Research Center of the Chinese University of Hong Kong-Nanjing University, Hong Kong, SAR China

**Keywords:** Hormone receptors, Osteoporosis

## Abstract

Adolescent idiopathic scoliosis (AIS) is a complex three-dimensional structural deformity of the spine with unknown etiology. Although leptin has been postulated as one of the etiologic factors in AIS, its effects on osteoblastic activity remain unknown. Herein, we conducted this study to investigate whether there are abnormal functional responses to leptin and abnormal expression of leptin receptor in AIS osteoblasts. *In vitro* assays were performed with osteoblasts isolated from 12 severe AIS girls and 6 non-AIS controls. The osteoblasts were exposed to different concentrations of leptin (0, 10, 100, 1000 ng/mL). The effects of leptin on cell proliferation, differentiation and mineralization were determined. Protein expressions of leptin receptor (LEP-R) under basal and osteogenic conditions were also evaluated by Western blot. Our results showed that leptin significantly stimulated osteoblasts from non-AIS subjects to proliferate, differentiate and mineralized. However, in the AIS group, the stimulatory effects of leptin on cell proliferation, differentiation, and mineralization were not observed. In addition, no statistically significant difference in the expression of leptin receptor under both basal and osteogenic conditions was found between AIS and control group. In conclusion, these findings might help to explain the low bone mass and deranged bone quality that is clinically associated with AIS girls.

## Introduction

Adolescent idiopathic scoliosis (AIS) is a complex three-dimensional structural deformity of the spine and its etiology remains unknown. It mainly occurs in girls between 11 to 14 years of age with a prevalence rate of 4%. In previous years, studies have reported associations between AIS and low body weight, tall stature, increased arm span, reduced body mass index, delayed menarche and decreased bone mass^1–4^. Special attention was paid to low bone mass as it was found to be a significant prognostic indicator for curve progression^5,6^. If left unattended, the status of low bone mass could persist into adulthood^7^. However, what mediates the observed characteristic anthropometric phenotypes, delayed pubertal development, abnormal skeletal growth and disturbed bone mineral homeostasis in AIS remains uncertain.

Leptin has been postulated as one of the etiologic factors of AIS because of its important physiological functions in neuro-osseous development affecting skeletal growth, the onset of puberty, energy expenditure and body composition. In addition, a number of *in vitro* functional studies lend support to direct anabolic effects of leptin on bone cells, including promotion of proliferation of osteoblasts^8^ and chondrocytes^9^, stimulation of differentiation and matrix mineralization^8,10^, induction of osteogenic differentiation and suppression of adipogenesis^11^, as well as inhibition of osteoclastogenesis and osteoclastic activity^8,12^. Recently, a large prospective cohort study reported that reductions in leptin, lean and fat mass were associated with an increased risk of scoliosis in adolescents, which provided supporting evidence for the potential link between leptin and AIS^13^. An animal study has also uncovered that elevated central leptin activity could increase the risk of developing scoliosis^14^. In our previous paper, we reported an abnormal leptin bioavailability with increased levels of the soluble leptin receptor (sOB-R) in AIS girls^15^, which was associated with suboptimal bone qualities including lower volumetric BMD in cortical bone and abnormal trabecular bone micro-architecture attributable to impaired osteoblast activities^16,17^. These results suggested a potential role of leptin in contributing to an abnormal osteoblastic activity in AIS.

Further to the clinical observations reported in previous publications, this study aimed to examine the effects of exogenous leptin on proliferation, differentiation, and mineralization in osteoblasts in primary culture isolated from bone biopsies of AIS patients, and to compare the effects with those on their non-AIS counterparts.

## Results

### Effect of leptin on cell proliferation of osteoblasts

**Figure 1:**
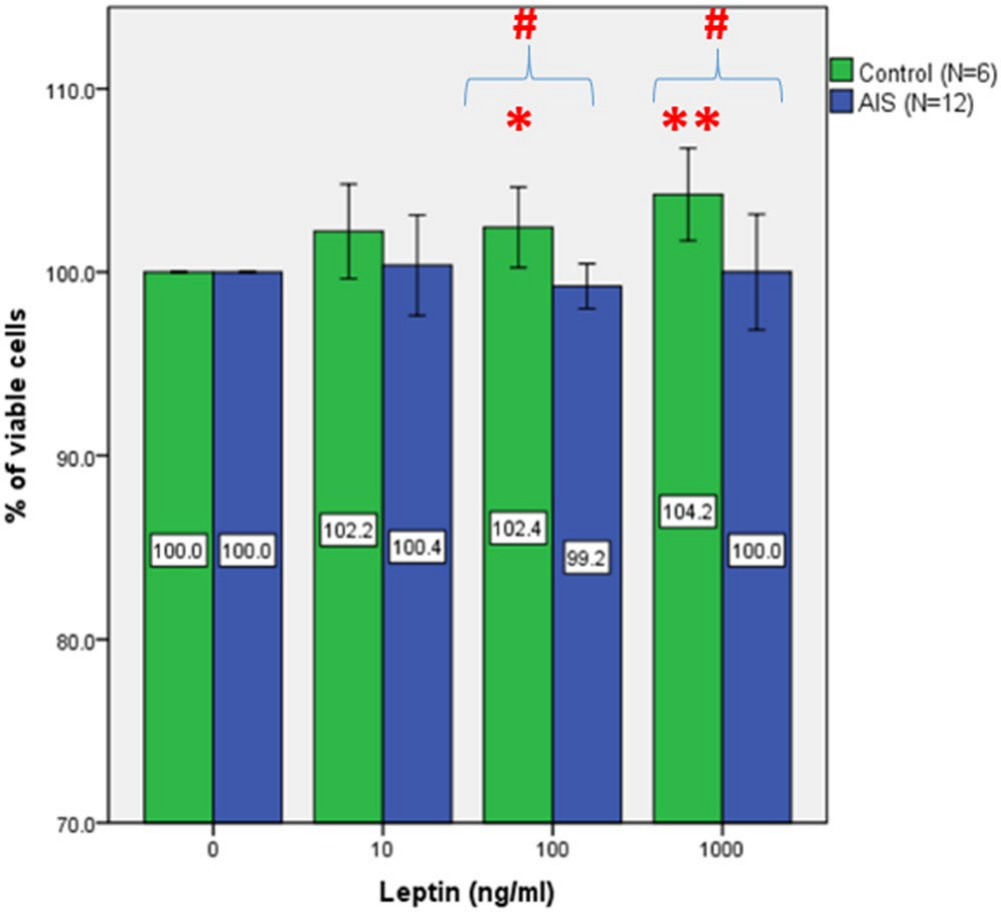
Effect of leptin on cell viability of human primary osteoblasts from AIS patients and control subjects. The primary osteoblasts from AIS patients showed no response in cell viability when treated with leptin at various concentrations for 72 hours. Cell viability was measured with the MTT assay. A representative example of 3 independent experiments. Each data point represents the mean of 5 replicate determinations ± SD. *p < 0.05; **p < 0.01, when compared with 0 ng/ml null treatment. ^#^p < 0.05; ^##^p < 0.01, when compared with control subjects.

After leptin treatment for 3 days, the proliferation of control osteoblasts was significantly stimulated in a dose-dependent manner (p < 0.01) (Fig. 1). The percentages of proliferation in controls were increased by 2.2%, 2.4%, and 4.2% at 10, 100, and 1000 ng/ml, respectively. Compared with the untreated group, the increase in percentage of proliferation was significant at 100 ng/ml (p < 0.05) and 1000 ng/ml (p < 0.01). However, the dose-dependent effect was not observed in the AIS osteoblasts (Fig. 1). There was no significant difference in the proliferation of AIS osteoblasts at any leptin concentration when compared with the untreated group. When the controls were compared with the AIS, the differences in proliferation at 100 ng/ml and 1000 ng/ml were statistically significant (p < 0.05).

### Effect of leptin on cell differentiation of osteoblasts

**Figure 2:**
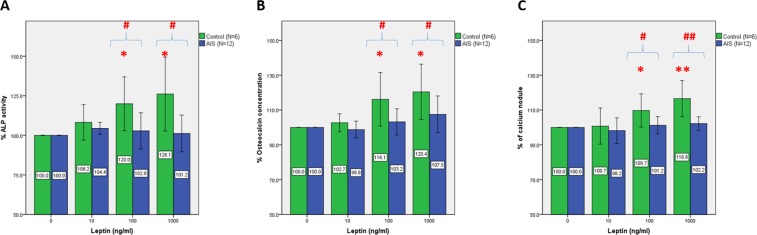
Effect of leptin on osteogenic differentiation and mineralization of human primary osteoblasts from AIS patients and control subjects. (**A**) Alkaline phosphatase (ALP) activity in the primary osteoblasts from AIS patients and non-AIS control subjects after leptin treatment for 14 days (0, 10, 100, 1000 ng/ml); (**B**) Osteocalcin concentration in culture medium collected from primary osteoblasts from AIS patients and non-AIS controls after leptin treatment for 35 days (0, 10, 100, 1000 ng/ml); (**C**) Quantification of the calcium nodule formed from primary human osteoblasts of AIS patients and non-AIS control subjects after leptin treatment for 35 days (0, 10, 100, 1000 ng/ml) by Von Kossa staining with image analysis. A representative example of 3 independent experiments. Each data point represents the mean of 4 replicate samples ± SD. *p < 0.05; **p < 0.01, when compared with 0 ng/ml null treatment. ^#^p < 0.05; ^##^ p < 0.01, when compared with control.

The ALP activities in the osteoblast cell lysates were measured after treatment with leptin for 14 days (Fig. 2A), with normalization using the total protein concentration. The controls showed enhanced ALP activity with a significant linear dose-dependent trend in response to increasing leptin concentrations (p < 0.05). The percentages of ALP activity in controls were increased by 8.2%, 20.0%, and 26.1% on day 14 (Fig. 2A) at the leptin concentrations of 10, 100, and 1000 ng/ml, respectively. The increase in percentage of ALP activity was significant at 100 ng/ml (p < 0.05) and 1000 ng/ml (p < 0.05). However, this dose-dependent effect was not observed in the AIS osteoblasts after 14 days of treatment. There were no significant differences in the ALP activity of AIS osteoblasts at any leptin concentration when compared with the untreated group. Comparing between the controls and AIS disclosed statistically significant differences in ALP activity at various leptin concentrations (p < 0.05).

Leptin stimulated the secretion of osteocalcin in control osteoblasts in a linear dose-dependent manner (p < 0.01) (Fig. 2B). The percentages of osteocalcin concentration in culture medium from control osteoblasts were increased by 2.7%, 16.1%, and 20.4% at the leptin concentrations of 10, 100, and 1000 ng/ml, respectively. The increase in percentage of osteocalcin concentration was significant at 100 ng/ml (p < 0.05) and 1000 ng/ml (p < 0.05). Although AIS osteoblasts showed a rising trend with increasing leptin concentrations, this linear relationship was only marginally significant (p = 0.057). There were no significant differences in the osteocalcin concentrations in culture medium from AIS osteoblasts. When comparing between the control and AIS, the differences in osteocalcin were statistically significant at 100 and 1000 ng/ml (p < 0.05).

### Effect of leptin on cell mineralization of osteoblasts

It was observed that control osteoblasts possessed a greater abundance of mineralized calcium nodules (Fig. 2C). Leptin stimulated the *in vitro* mineralization of control osteoblasts in a dose-dependent manner with a significant linear trend (p < 0.01). The percentages of mineralization in controls were increased by 0.7%, 9.7%, and 16.6% at the leptin concentrations of 10, 100, and 1000 ng/ml, respectively. Comparing with the untreated group indicated a significant increase in the percentage of mineralization at 100 ng/ml (p < 0.05) and 1000 ng/ml (p < 0.01). However, the dose-dependent effect was not observed in the AIS osteoblasts (Fig. 2C) (p = 0.305). There were no significant differences in the mineralization of AIS osteoblasts at any leptin concentration. A comparison between the control and AIS revealed a significant difference in calcium nodule formation at 100 ng/ml (p < 0.05) and 1000 ng/ml (p < 0.01).

### Expression of leptin receptor in osteoblasts

**Figure 3:**
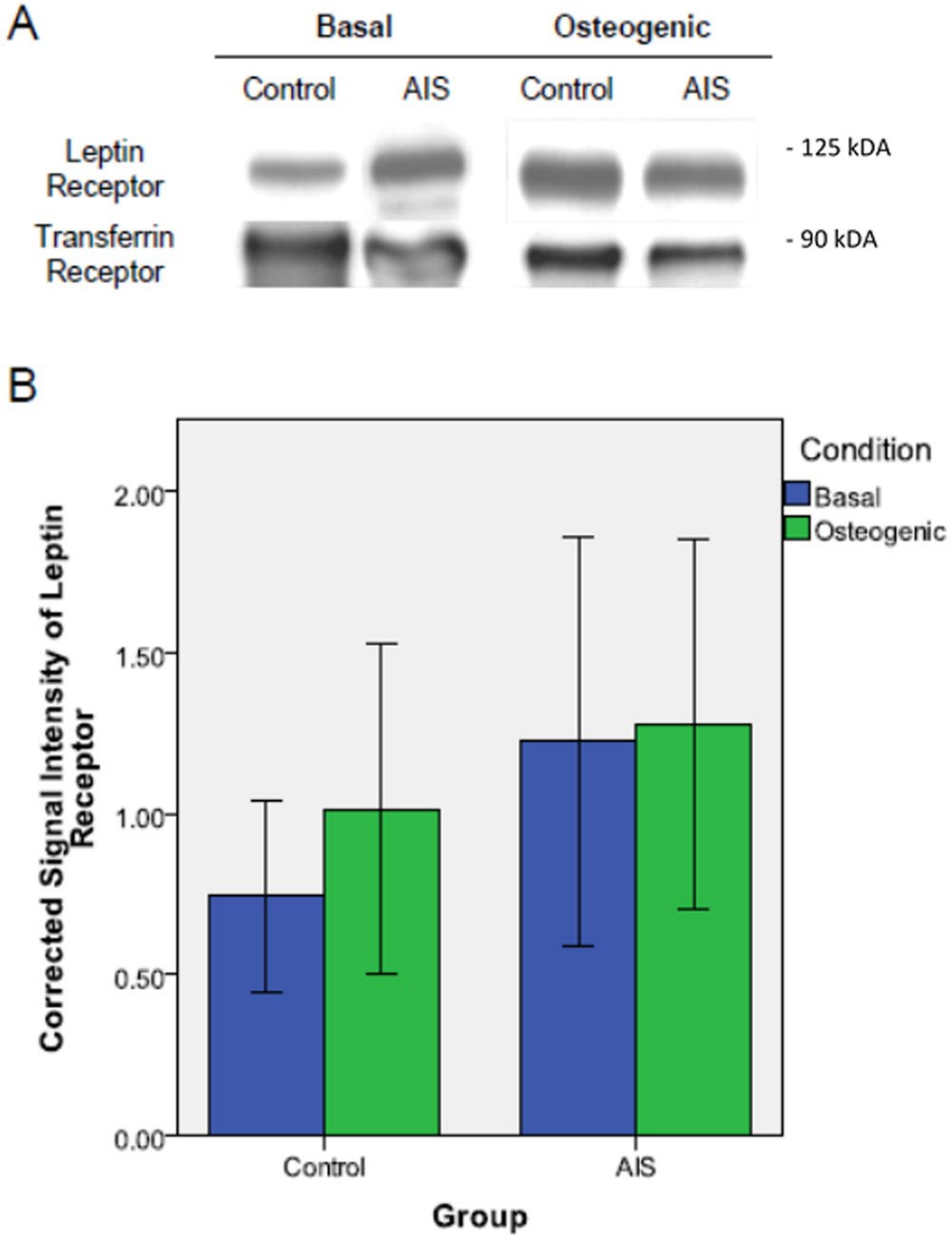
Leptin receptor expression level in control and AIS osteoblasts. (**A**) Representative Western blot was used to detect leptin receptors under basal and osteogenic conditions in control and AIS osteoblasts with transferrin receptor as loading control. (**B**) Corrected signal intensity of leptin receptor under basal and osteogenic conditions in control and AIS osteoblasts. A higher corrected signal intensity was observed in the AIS group at both conditions when compared with the controls. No significant difference was observed between the two conditions when compared within the AIS nor control groups.

Representative protein bands of leptin receptor in control and AIS osteoblasts under basal and osteogenic conditions exhibited expression of the functional long form of leptin receptor (Fig. 3A). Upon quantification of the signal intensity of leptin receptor, no difference was observed between groups under both conditions (basal: p = 0.275; osteogenic: p = 0.518) and between different conditions within groups (control: p = 0.289; AIS: p = 0.848) (Fig. 3B). However, it was noted that the expression of leptin receptor in control increased notably under osteogenic condition (difference = 36.0% compared with basal condition), while in AIS osteoblasts the expression of leptin receptor remained unchanged.

## Discussion

In addition to our previous reports on anthropometric abnormalities and abnormal body composition in AIS girls, it was discovered in our current study that leptin could dose-dependently enhance proliferation, differentiation and mineralization of normal human osteoblasts, but similar effects were not observed in AIS osteoblasts. When a comparison between the normal controls and AIS osteoblasts was made, there were statistically significant differences in proliferation, differentiation and mineralization in response to leptin at 100 ng/mL and 1000 ng/mL concentration of leptin. However, no significant differences in the expression of leptin receptor under basal and osteogenic conditions were found between AIS and control group.

Previous studies have reported that osteopenia in AIS girls is systemic in nature and could affect the whole body including the spine, hip, distal radius, distal tibia, and calcaneus^2,3,18^. Based on the clinical association, we postulated that bone cells from AIS girls and non-AIS control subjects might respond differently to leptin. In the osteoblasts isolated from non-AIS controls, significant dose-dependent effects were observed in the functional responses to increasing leptin concentrations. Similar to the previous study, treatment of primary human osteoblasts from osteoarthritis patients with 0 and 100 ng/ml of leptin for 35 days on primary human osteoblasts from osteoarthritic patients was found to enhance mineralization from 8% to 42% of the mineralized surface^10^. In another study using primary human osteoblasts, it was demonstrated that leptin could significantly increase the percentage of mineralized surface from a plateau of 12–28% and 33% of mineralized surface on days 28 and 35, respectively^8^. Contrariwise, primary osteoblasts isolated from AIS patients showed no significant responses to leptin in proliferation, differentiation and mineralization. This may imply that there may be functional impairment in the response of AIS osteoblasts to leptin. The decreased cell proliferative response to leptin in AIS could be associated with a diminished number of osteoblasts which are vitally important for collagen synthesis and bone formation. In addition, the abnormal response in ALP activity to leptin treatment, together with the lower baseline level of osteocalcin and decreased secretion in response to leptin, signify possible delayed and/or decreased differentiation into mature osteoblasts, which eventually affects bone mineralization process. These results were justified with significantly lower calcium nodules and an abated mineralization response to leptin detected in AIS osteoblasts.

Leptin has also been shown to down-regulate bone resorption process by inhibiting osteoclastogenesis and osteoclast activity^8,12,19^ Given the importance of leptin in directly stimulating bone formation and inhibiting bone resorption, the observed hyposensitivity to leptin in AIS could lead to an imbalance in these normally coupled processes, resulting in down-regulated bone formation and up-regulated bone resorption, and the low bone mass and deranged bone quality that were found in AIS girls. Given the observed mitigated functional responses to leptin, it is logical to suspect a downregulated expression of functional leptin receptor on AIS osteoblasts. However, our study showed no significant difference in the expression of functional leptin receptor between AIS and control osteoblasts, under both basal and osteogenic conditions. This finding, together with the possible absence of induction of leptin receptor under osteogenic stimulation, suggested another possibility that AIS osteoblasts could have attenuated intrinsic cellular response to extracellular stimuli, thus leading to reduced proliferation, differentiation, and mineralization upon application of extrinsic stimuli such as osteogenic medium, leptin and other factors such as melatonin^20^. Leptin signaling under normal conditions is subject to negative feedback regulation and leptin challenge could reduce the expression of leptin receptors on the cells. This speculation is supported by a study showing that the expression of leptin and leptin receptor in the MSCs of AIS group were not responsive to changes in exogenous leptin levels^21^, which is similar in AIS osteoblasts. Additionally, the abnormal responses to leptin might not be due to difference in expression of functional leptin receptor but involving other possibilities, e.g. defects in the leptin downstream-signaling pathways or dysfunctional leptin receptor. Previous studies have provided some evidence suggesting that the Jak/Stat signaling pathway might be dysfunctional leading to hyposensitivity to leptin in AIS^21^. However, further studies are still warranted.

For ethical and practical reasons, it is very difficult to obtain bone biopsies for control subjects. In this study, the age range of the control group was generally larger than that of the AIS patients, and included both genders, while AIS patients only included females. However, there was no statistical significance difference in age when between the AIS and controls (p = 0.917). Additionally, the bone biopsies from controls were also obtained from different anatomical locations as compared with AIS patients. However, as shown in another study, the functionally active long isoform of leptin receptor (OB-Rb) did not change with age or sex^22^. It is uncertain whether osteoblasts isolated from different anatomical locations express the leptin receptor to different levels and have a uniform response to leptin signaling^23^, but this seems unlikely given that the isolated osteoblasts were cultured an identical, controlled environment compared to anatomical locations where varied oxygen and nutrient levels, amount of fat tissue, and weight bearing effect could exist *in vivo*.

In conclusion, this study has clearly demonstrated the abnormal responses to proliferation, differentiation and mineralization in AIS osteoblasts when exposed to various concentrations of leptin. The hyposensitivity to leptin is a plausible explanation for the abnormally low bone mineral density observed in the AIS patients. Hence, further studies on the interaction between leptin and osteoblasts may elucidate the possible link in the etiopathogenesis of AIS.

## Materials and Methods

### Ethical approval

All human cell harvesting was performed with the approval and in compliance with guidelines and regulations of the Joint Chinese University of Hong Kong - New Territories East Cluster Clinical Research Ethics Committee (CREC), Hong Kong (CRE 2007.123 and CRE 2010.066). Informed written consent was obtained from all of the subjects as well as from their legal guardians before enrollment into this study. In addition, all aspects of this research were performed in accordance with the relevant guidelines and regulations.

### Tissue collection

Twelve girls (aged 13 to 19, mean age: 16.2 ± 2.2) with severe AIS undergoing posterior spinal instrumentation and fusion surgery were recruited from the Joint Scoliosis Research Center of the Chinese University of Hong Kong and Nanjing University. Bone biopsies were collected from the iliac crest autograft bone harvesting site in the AIS patients (Table 1). In addition, six non-scoliotic normal controls (4 females and 2 males) aged between 0.7 to 23 years (mean age: 11.5 ± 11.3) were recruited^16,20^. For the controls, cancellous bone biopsies were obtained from bone tunnels of patients undergoing anterior cruciate ligament reconstruction (1 case) or the non-affected sites of patients with degenerative spine undergoing spinal fusion surgery (5 cases) (Table 2).

**Table 1:** Clinical data of patients with AIS recruited for the present investigation.

Case no.	Diagnosis	Curve Pattern	Gender	Age at surgery (yr)	Cobb Angle (^o^)
A1	AIS	R/L Double Curve	F	18.9	62/89
A2	AIS	L/R/L Triple curve	F	14.3	32/61/35
A3	AIS	L/R/L Triple curve	F	13.3	37/77/44
A4	AIS	L/R/L Triple curve	F	16.8	37/69/40
A5	AIS	R Thoracic curve	F	17.1	60
A6	AIS	R/L Double curve	F	17.5	69/51
A7	AIS	L/R Double curve	F	16.2	60/80
A8	AIS	L/R/L Triple curve	F	14.3	46/81/66
A9	AIS	L/R/L Triple curve	F	19.5	45/50/11
A10	AIS	L/R/L Triple curve	F	13.9	38/86/55
A11	AIS	L/R/L Triple curve	F	18.9	50/67/30
A12	AIS	L/R/L Triple curve	F	13.7	61/103/48

AIS, adolescent idiopathic scoliosis; L, left; R, right; F, female.

**Table 2:** Clinical data of control patients recruited for the present investigation.

Case no.	Type of surgery during which intraoperative bone biopsies were obtained	Site of bone biopsy	Curve Pattern	Gender	Age at surgery (yr)
N1	ACL reconstruction	Distal femur	Nil	M	18.9
N2	Developmental Dysplasia of Hip	Iliac crest	Nil	F	14.3
N3	Developmental Dysplasia of Hip	Iliac crest	Nil	F	13.3
N4	Exstrophy of Bladder	Iliac crest	Nil	M	16.8
N5	Dental Extraction	Right mandible	Nil	F	17.1
N6	Dental Extraction	Right mandible	Nil	F	17.5

ACL, Anterior cruciate ligaments; F, female; M, Male.

### Osteoblast culture

Trabecular bones from the biopsies were cut and minced into small pieces with a sharp bone cutter under sterile conditions. The fragments were plated onto a 6-well culture plate (Corning, New York, NY, USA) and cultured in DMEM (Invitrogen) supplemented with 10% fetal bovine serum (FBS) (Invitrogen) and 1% penicillin-streptomycin-neomycin (PSN) antibiotic mixture (Invitrogen, Carlsbad, NM, USA). The culture was maintained at 37 °C in a humidified atmosphere of 5% CO_2_. The medium was renewed every 2–3 days. Osteoblasts were harvested by the addition of 0.25% trypsin (Invitrogen) upon reaching 90% confluence for subculture. The second passage of cells was then used for the following assessments.

### Assay of cell viability

The effect of leptin on the proliferation of normal human osteoblasts and AIS osteoblasts was analyzed using the MTT assay, as previously described^24^. In brief, the osteoblasts were trypsinized and seeded into a 96-well tissue culture plate at a concentration of 2000 cells/well. The cells were allowed to attach for 24 h. Then, the medium was replaced and cultured for 3 days in various concentrations (0, 10, 100, and 1000 ng/ml) of leptin, with daily renewal of the medium and leptin^8,20^. On day 3, the cells were rinsed with phosphate-buffered saline (PBS) and MTT solution was added. After incubation at 37 °C for 4 h in the dark, the MTT solution was carefully removed. The formazan in the cells was released by the addition of DMSO at room temperature. Absorbance was measured at 570 nm using a microplate reader (Biotek, Winooski, USA). All measurements were performed with 5 replicates and in 3 independent experiments.

### Assay of alkaline phosphatase (ALP) activity

ALP activity was determined using p-nitrophenyl phosphate (p-NPP) as substrate^25^. In brief, osteoblasts were plated (10,000 cells per well) in a 12-well culture plate. The cells were cultured in basal medium until confluence. Thereafter, the cells were treated with osteogenic medium (10 mM l-ascorbic acid, 10 mM β-glycerophosphate, 100 nM dexamethasone and basal α-MEM) containing various concentrations (0, 10, 100, and 1000 ng/ml) of leptin for 14 consecutive days with renewal of medium and leptin every 2 to 3 days. On day 14, the medium was removed and rinsed with sterile PBS. The cells were then lysed with repeated freeze and thaw cycles in the presence of DEA lysis buffer. To determine ALP activity, 20 μl of the supernatant was transferred to a 96-well plate, and 130 μl of PNPP substrate (Sigma, St. Louis, USA) was added. The reaction mixture was stopped by adding 50 μl of 3 M NaOH. The absorbance was measured at 405 nm using a microplate reader (Biotek, Winooski, USA). The amount of PNP formed was determined from a standard curve constructed by measuring a range of PNPP standards. The results were normalized by the total protein levels in the lysate supernatants measured with bicinchoninic acid (BCA) protein assay (Pierce, Waltham, USA).

### Osteocalcin ELISA

Cells were plated at a density of 10,000 cells per well in a 12-well culture plate. The cells were cultured in basal medium until 80% confluence. Thereafter, the cells were treated with osteogenic medium containing various concentrations (0, 10, 100, 1000 ng/ml) of leptin for 35 consecutive days with renewal of medium and leptin every 2 to 3 days. The culture medium was collected on day 10, 17, 24, 31, and 35 and stored at −80 °C until analysis. Osteocalcin levels were measured with ELISA that is specific for human intact osteocalcin (Takara Bio Inc., Mountain View, California) following a standard protocol recommended by the manufacturer.

### Von kossa staining

The degree of mineralization was assayed by von Kossa staining on day 35. The cells were washed with PBS for three times. The cells were then fixed in 4% paraformaldehyde for 15 minutes at room temperature. The fixed cells were washed three times with distilled water. Afterwards, 500 μl 5% silver nitrate solution was added and the fixed cells were exposed under light for 1 hour. Then, 500 μl 5% sodium thiosulfate solution was added and incubated for 5 minutes. The samples were washed once with distilled water and photographed. The stained pictures were analyzed with image analysis (Image-Pro Plus 6.0, Media Cybernetics, Inc., Rockville, USA) to quantify the intensities of the stains. Three independent experiments were conducted and all measurements were performed with 5 replicates.

### Western blotting

Total proteins were collected with cold hypotonic buffer plus protease inhibitors from cells with and without osteogenic induction. Equal amounts (30 µg) of each sample were resolved on 10% SDS-PAGE gel and transferred to nitrocellulose membrane (Hybond-ECL; GE Healthcare, Bucks, UK). After blocking with 5% nonfat dry milk for 1 h at room temperature, membranes were incubated with rabbit IgG anti-human leptin receptor primary antibody (Santa Cruz, Dallas, USA) (1:500 in 3% BSA solution) or rabbit IgG anti-human transferrin receptor primary antibody (Cell Signaling Technology, Danvers, USA) (1:1000 in 3% BSA solution) overnight at 4 °C with agitation. Antibody-specific labeling was revealed by incubation with a horseradish peroxidase (HRP) conjugated goat anti-rabbit secondary antibody (Santa Cruz, Dallas, USA) (1:5000 in 5% skimmed milk blocking solution) for 1 h and visualized with the ECL Western Blotting Detection Reagents (GE Healthcare, Bucks, UK) on X-ray film (Fujifilm, Tokyo, Japan).

### Statistical analysis

All data were expressed as mean ± standard deviation (SD). Student’s t test was used to compare differences between the AIS and control groups, and one-way ANOVA with post hoc analysis was used to compare differences when there were more than two groups. SPSS 16.0 for Windows (SPSS Inc., Chicago, IL) was used for all statistical analyses. A *p* value < 0.05 was considered as statistically significant.
